# Supplementary material for: Room-temperature stabilizing strongly competing ferrielectric and antiferroelectric phases in PbZrO3 by strain-mediated phase separation
Source: Nat Commun. 2024 Apr 23;15:3438. doi: 10.1038/s41467-024-47776-6 (PMC11039770; doi:10.1038/s41467-024-47776-6)
Supplement: Supplementary file 1 — Supplementary Information [file 41467_2024_47776_MOESM1_ESM.pdf]

Supporting Materials of “Room-temperature Stablizing Strongly Competing Ferrielectric and Antiferroelectric phases in $PbZrO_3$ by Strain Mediated Phase Separation”

Ziyi Yu[1, 2], Ningbo Fan[3], Zhengqian Fu[1 *], Biao He[1], Shiguang Yan[1], Henghui Cai[1], Xuefeng Chen[1], Linlin Zhang[1], Yuanyuan Zhang[4], Bin Xu[3], Genshui Wang[1 *] and Fangfang Xu[1, 2 *]

Affiliations:

[1] State Key Laboratory of High Performance Ceramics and Superfine Microstructures & The Key Lab of Inorganic Functional Materials and Devices, Shanghai Institute of Ceramics, Chinese Academy of Sciences, Shanghai 200050, China

[2] School of Physical Science and Technology, ShanghaiTech University, Shanghai 201210, China

[3] Jiangsu Key Laboratory of Thin Films, School of Physical Science and Technology, Soochow University, Suzhou 215006, China

[4] Key Laboratory of Polar Materials and Devices, Ministry of Education, Department of Electronic Science, East China Normal University, Shanghai, 200241, China

*Correspondence to: fmail600@mail.sic.ac.cn (Z. F.); genshuiwang@mail.sic.ac.cn (G. W.); ffxu@mail.sic.ac.cn (F. X.).

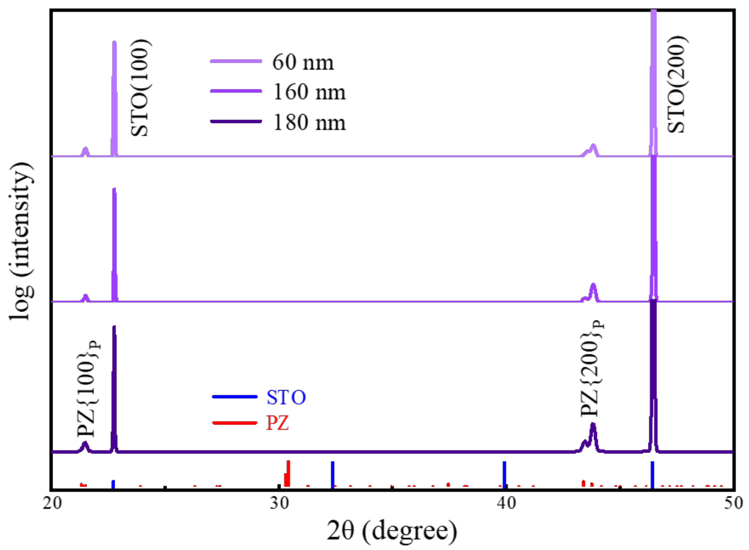

Fig. S1. The X-ray diffraction (XRD) patterns of PZ/STO thin films with different thickness exhibit obvious diffraction peaks of PZ {100}P, indicating well-epitaxial layer quality.

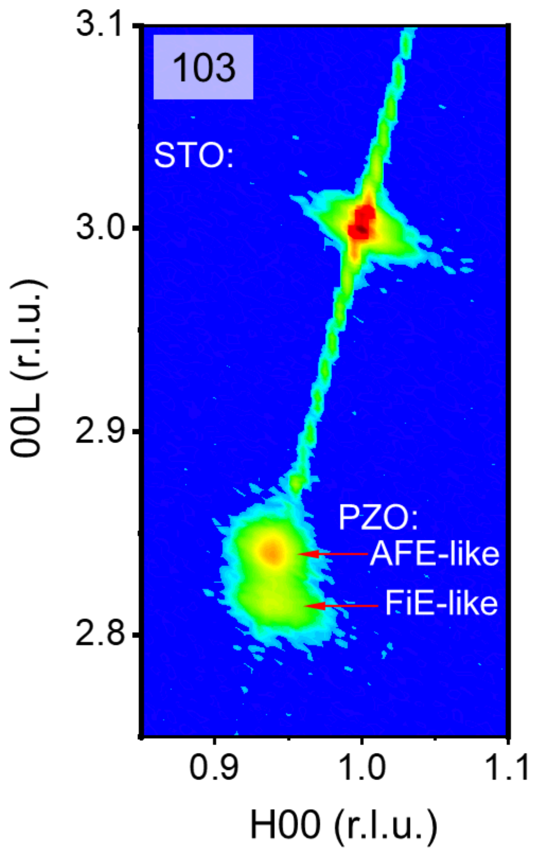

Fig. S2. Logarithmic reciprocal maps in the H0L scattering plane around the $(103)_c$ reflections for the 180 nm $PbZrO_3$ thin film directly grown on $SrTiO_3$ substrate. (Intensities from low to high: blue-green-yellow-red-brown)

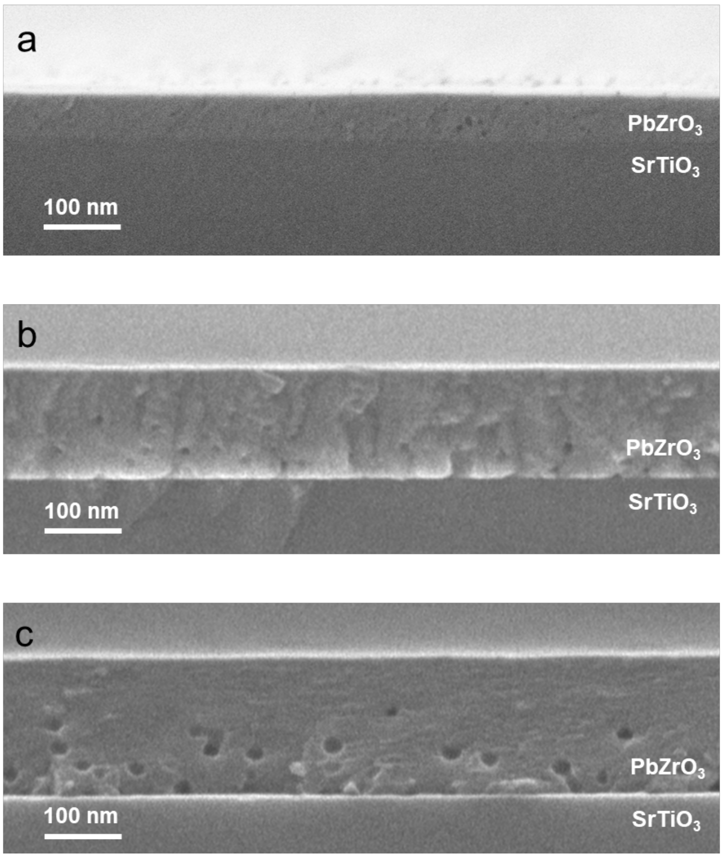

Fig. S3. The cross-sectional SEM images of $PbZrO_3/SrTiO_3$ thin films with different film thickness. (a) is the thickness of ~60 nm, (b) ~160 and (c) ~180 nm, respectively.

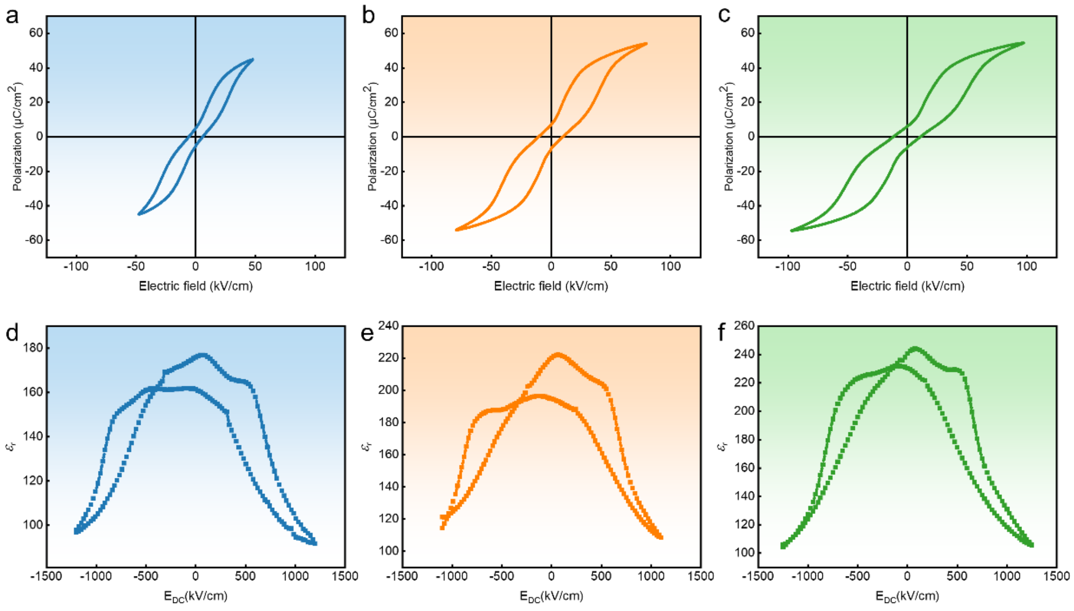

Fig. S4. Hysteresis loops (a-c) and the ε_r-E curves (d-f) for the PZ thin films with different thickness. (a, d) is the thickness of 60 nm, (b, c) is of 160 nm and (c, f) is of 180 nm, respectively

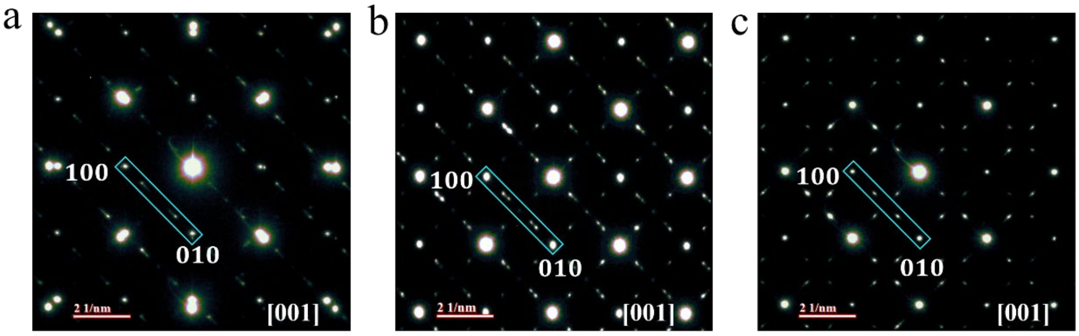

Fig. S5. The electron diffraction patterns of PZ films with thickness of 60 (a), 160 (b) and 180 nm (c), respectively. The blue rectangles labels the area of magnified SAED patterns in Fig. 2a~2c. The appearance of the streaking features in diffraction patterns implies the presence of a high density of phase boundaries.

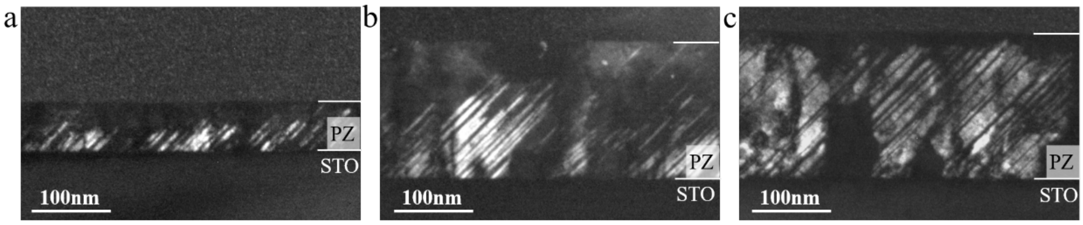

Fig. S6. The cross-sectional dark-field images of films with thickness of 60 (a), 160 (b) and 180 nm (c), respectively. The bright contrast refers to the FiE phase.

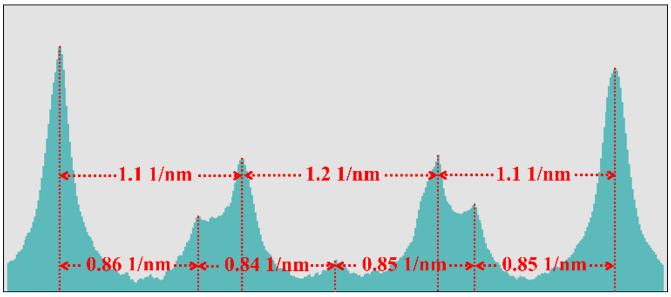

Fig. S7. Intensity profile of the reflections in the inset of Fig. 2d showing different modulation periods for the mixed phases.

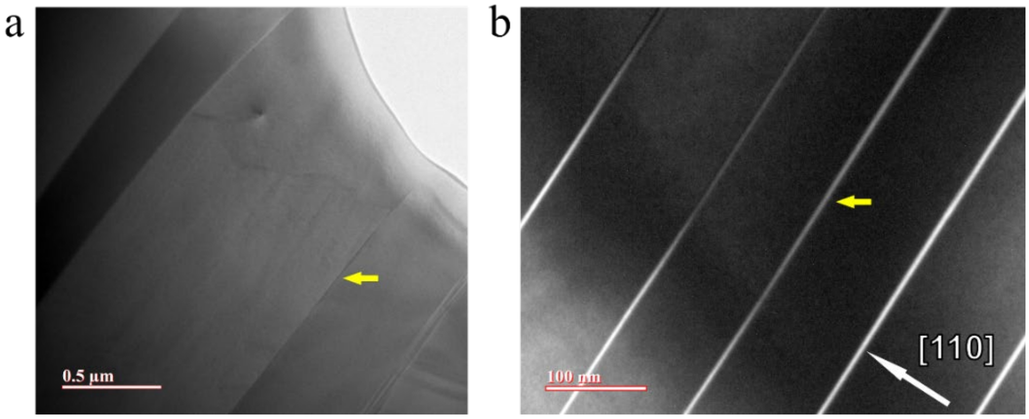

Fig. S8. TEM observation of PZ ceramics. (a) Bright-field image of AFE domains; (b) Dark-field image of antiphase domains where the parallel APBs locate on the (110) plane. The yellow arrows point out representatively the AFE domain boundaries in (a) and antiphase domain boundaries in (b).

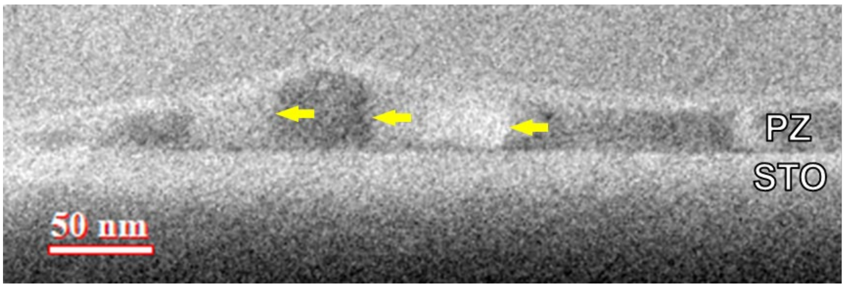

Fig. S9. HAADF-STEM image of the PZ/STO thin films. The yellow arrows point out the position of domain walls.

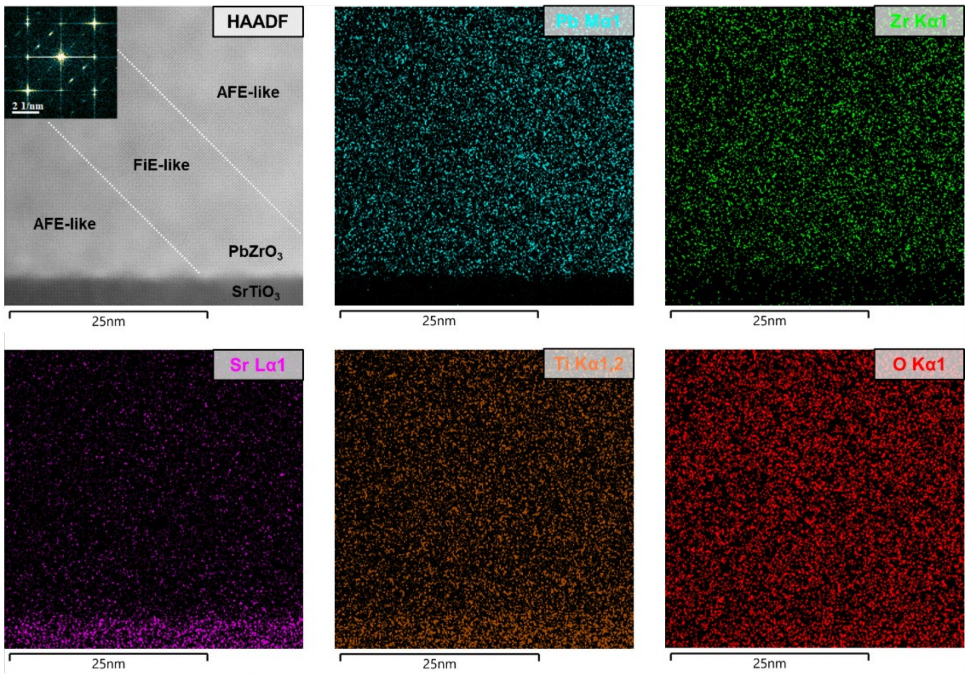

Fig. S10. EDS mapping of the PZ thin film with thickness of 180nm.

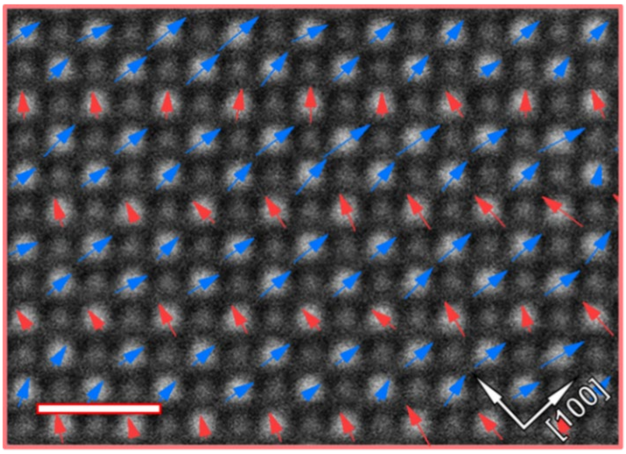

Fig. S11. [001]-projected HAADF-STEM images with superimposed mapping of Pb displacements (see arrows) in FiE phase in the present PZ thin film. The scale bar refers to 1 nm.

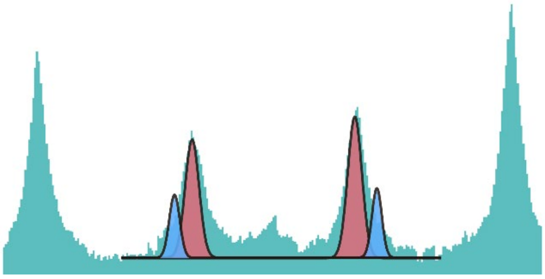

Fig. S12. The intensity profiles of the magnified SAED patterns in Fig. 2c. approximately overlayed by the fitting peaks (FiE: red; AFE: blue). The volume fraction was obtained by: vol%= the area of FiE/(the area of FiE+the area of AFE).

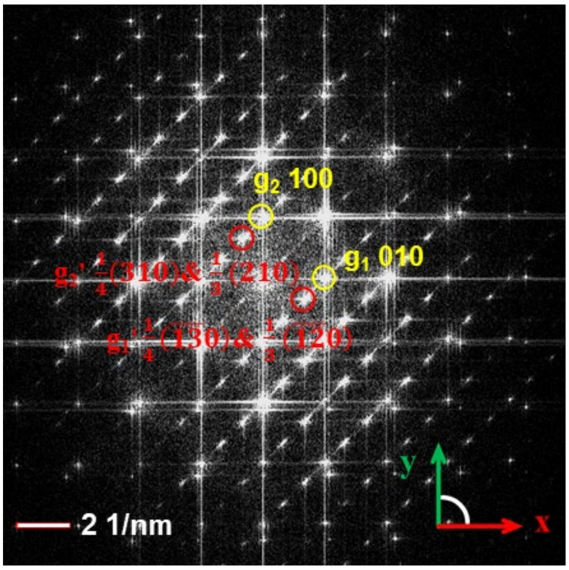

Fig. S13. Fourier transform of image in Fig. 3a. The $(0\bar{1}0)$ and (100) lattice fringes are selected to characterize the local strain compared with the PZ and STO substrate;

the center of $1/4\,(\bar{1}\bar{3}0)$ and $1/3\,(\bar{1}\bar{2}0)$, $1/4\,(310)$ and $1/3\,(210)$ are selected to characterize the local strain among mixed phases in PZ. The position and size of Gaussian masks are marked by red and yellow circles.

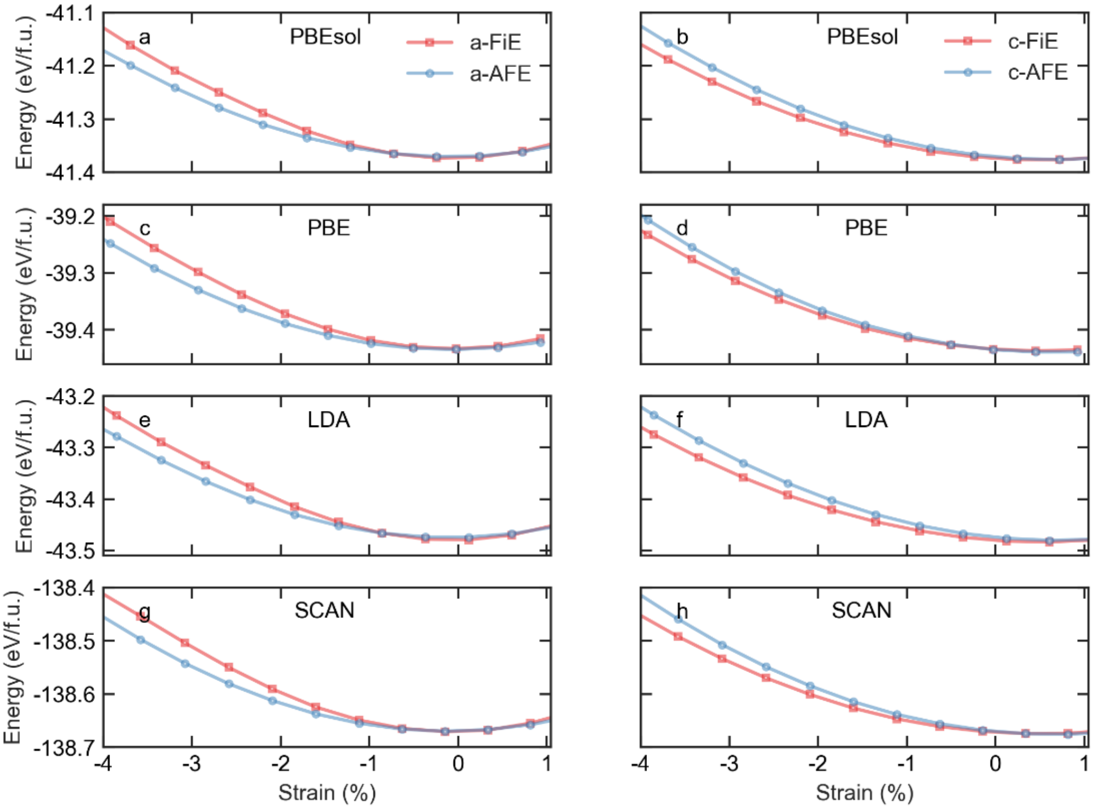

Fig. S14. Comparison of DFT predicted stabilities of the AFE and FiE phases, computed with various exchange-correlation funcionals. Total energies of the FiE and AFE phases as a function of epitaxial biaxial strain in (a) (100) plane and (b) in (001) plane with PBEsol, (c), (d) ibid with PBE, (e), (f) ibid with LDA, and (g), (h) ibid with SCAN.
